# Case report: A nodular lesion in the ventral region of the neck in the rat as a starting point for considerations on differential diagnosis

**DOI:** 10.3389/fvets.2024.1472317

**Published:** 2024-09-12

**Authors:** Agata Godlewska, Izabella Dolka, Ilona Borowczak, Ewa Chomutowska, Mirosław Przeworski, Katarzyna Różycka, Karolina Barszcz

**Affiliations:** ^1^Department of Morphological Sciences, Institute of Veterinary Medicine, Warsaw University of Life Sciences (SGGW), Warszawa, Poland; ^2^EDINA Veterinary Clinic PulsVet 24h, Warsaw, Poland; ^3^Department of Pathology and Veterinary Diagnostics, Institute of Veterinary Medicine, Warsaw University of Life Sciences (SGGW), Warsaw, Poland; ^4^Veterinary Clinic Forestvet, Warsaw, Poland; ^5^Veterinary Clinic ANIMAL.MED, Gdańsk, Poland

**Keywords:** pet rat, poorly differentiated sarcoma, tumor, ventral neck, differential diagnosis

## Abstract

The purpose of this case report is to present a poorly differentiated sarcoma in a pet rat. A veterinarian detected a small-sized nodular lesion in the ventral region of the neck during a follow-up visit related to another ailment. The anatomical structures found in the neck region in the rat and the differential diagnosis when deformities are palpated in this body part are discussed in detail. The patient underwent a total of four surgical procedures, as well as radiotherapy and chemotherapy. The rat survived in good condition for 144 days after finding the tumor.

## Introduction

When a nodular lesion is found on palpation in the neck region in a rat, it is essential to consider all possible differential diagnoses. It is the authors’ experience that it usually appears to be a tumor or an abscess, but it may be difficult to say with certainty where they originate from. Tumors of the mammary gland along with galactocele, salivary gland tumors and inflammation, enlarged lymph nodes in the course of lymphoid neoplasms, tumors of the thyroid and parathyroid glands, as well as tumors originating from the connective tissue, nerves, and vessels can be expected to appear in this region. When it comes to abscesses, these are usually subcutaneous deformities but may also arise in deeper tissues and organs of the neck (1, 2).

## Case description

**Figure 1:**
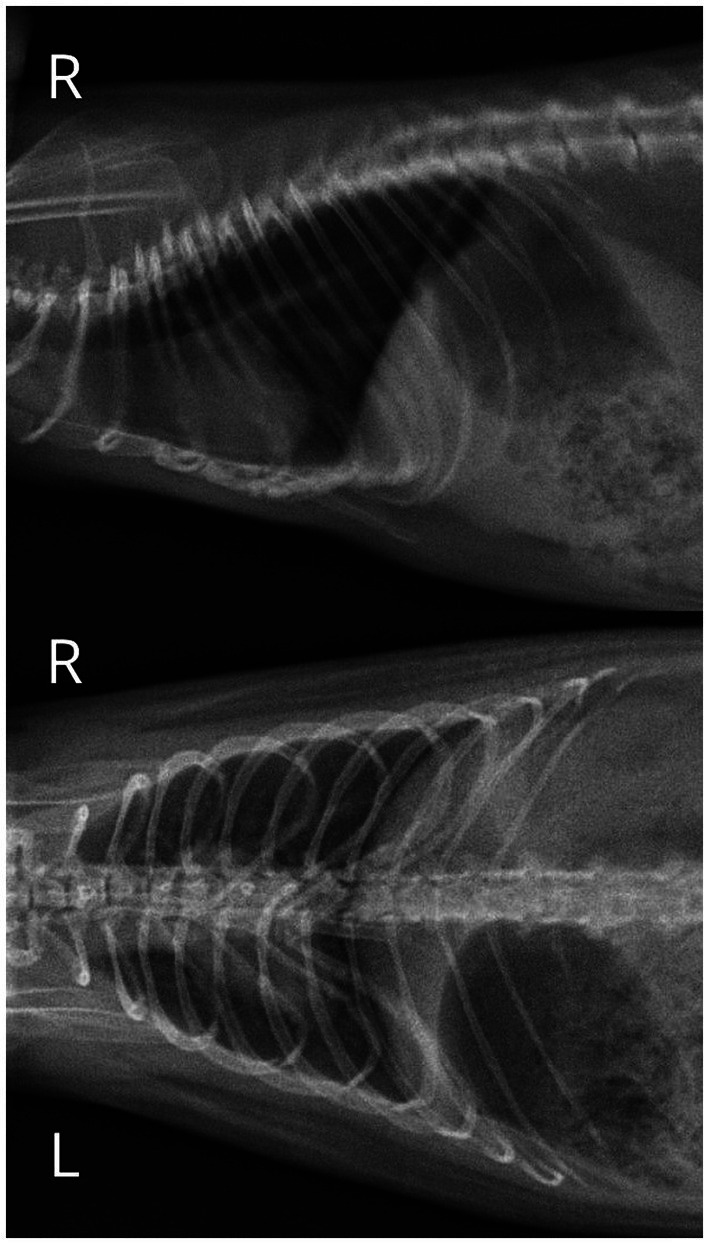
Chest radiographs showing a slight increase in lung opacity, diffuse and bilateral bronchial pattern with a slight loss of distinction of the heart contour, suggestive of pneumonia.

The patient was an 18-month-old male pet rat (*Rattus norvegicus* f. *domestica*) brought in for a follow-up visit associated with treatment for diabetes mellitus that was recognized 6 months earlier. To date, glucose levels had been controlled by subcutaneous insulin administration. The owner measured the glucose twice daily and adjusted the dose according to the glucose level (1–3 IU 2x daily). On clinical examination, the rat was obese (BCS 5/5) (3), with a reduction of muscle mass in the pelvic limbs, typical of older males (4). A well-demarcated, nodular lesion of approximately 4 mm in diameter was palpable in the left ventral neck region. It doubled in size over the following 2 weeks. There were no symptoms directly related to its presence. A blood sample was taken under isoflurane anesthesia. The results showed slightly elevated GLDH, ALT levels, and monocyte count, which was not considered a contraindication to general anesthesia (Table 1). In the authors’ opinion, the upper limit of urea is higher, and the lower limit of alpha-amylase is lower in rats than that given by Giknis and Clifford (5), so the results were deemed correct. Chest radiographs (Figure 1), echocardiography, and abdominal ultrasound were also performed. Chest radiographs showed a slight increase in lung opacity and a diffuse bilateral bronchial pattern with a slight loss of distinction of the heart contour, which was suggestive of pneumonia. There were no signs of metastases. Echocardiography showed left ventricular lumen dilatation with no impairment of systolic function, with a slight left atrial lumen dilatation and a slight aortic valve regurgitation. The abdominal ultrasound was unremarkable.

**Table 1:** Blood results and reference ranges.

Parameter	Blood results before the first surgery	Blood results before the oncology consult	Reference range according to Giknis and Clifford (5), to the authors*
RBC	9.42	8.94	7.62–9.99 T/L
Ht	47.2	47.4	38.5–52%
Hb	16.1	15.5	13.6–17.4 g/dL
MCV	50.1	53	46.3–56.2 fl
MCH	17.1	17.3	16.3–19.5 pg
MCHC	34.1	32.7	31.9–38.5 g/dL
WBC	5.29	5.2	1.98–11.06 G/L
Neutrophils	13.6	15.7	9–49.3%
Lymphocytes	76.6	72.7	44.7–87.1%
Monocytes	8.7	10.6	1–3.6%
Eosinophils	0.9	0.8	0.4–4%
Basophils	0.2	0.2	0–0.6%
PLT	841	1,137	574–1,253 G/L
ALT (GPT)	57.6	37.4	19–48 U/L
AST (GOT)	73.6	98.5	63–175 U/L
Alpha-amylase	577.1	435.7	1,223–2,109 U/L
TP	6.68	6.51	5.6–7.6 g/dL
CK	79	428	460–1,230
GLDH	39.6	15.2	0–17 U/L*
Creatinine	0.4	0.42	0.3–0.5 mg/dL
Urea	36.06	23.94	10.07–20 mg/dL
Glucose	135.5	457.2	106–184 mg/dL

* indicates, where the range level of GLDH is given.

The lesion was removed along with a part of the left mandibular salivary gland and an adjacent lymph node. Histopathological examination revealed malignant myoepithelioma of the mandibular salivary gland. It was infiltrated by poorly circumscribed and nonencapsulated nodular neoplasm, composed of spindeloid neoplastic cells arranged in interwoven irregular bundles without distinct orientation. They infiltrated between acini and glandular structures. The neoplastic cells had indistinct cell borders, a moderate amount of eosinophilic cytoplasm, and one spindeloid to elongated hyperchromatic nucleus. Mild to moderate anisocytosis and anisokaryosis was seen. Neoplastic cells were present at the sample’s margins. The lymph node was unremarkable.

The patient was referred for an oncology consultation. Therefore, a blood test was repeated. This time, hyperglycemia was noted along with monocytosis (Table 1), and the insulin dose was adjusted. It was decided to implement radiotherapy in a regimen of four fractions of 5Gy once a week.

**Figure 2:**
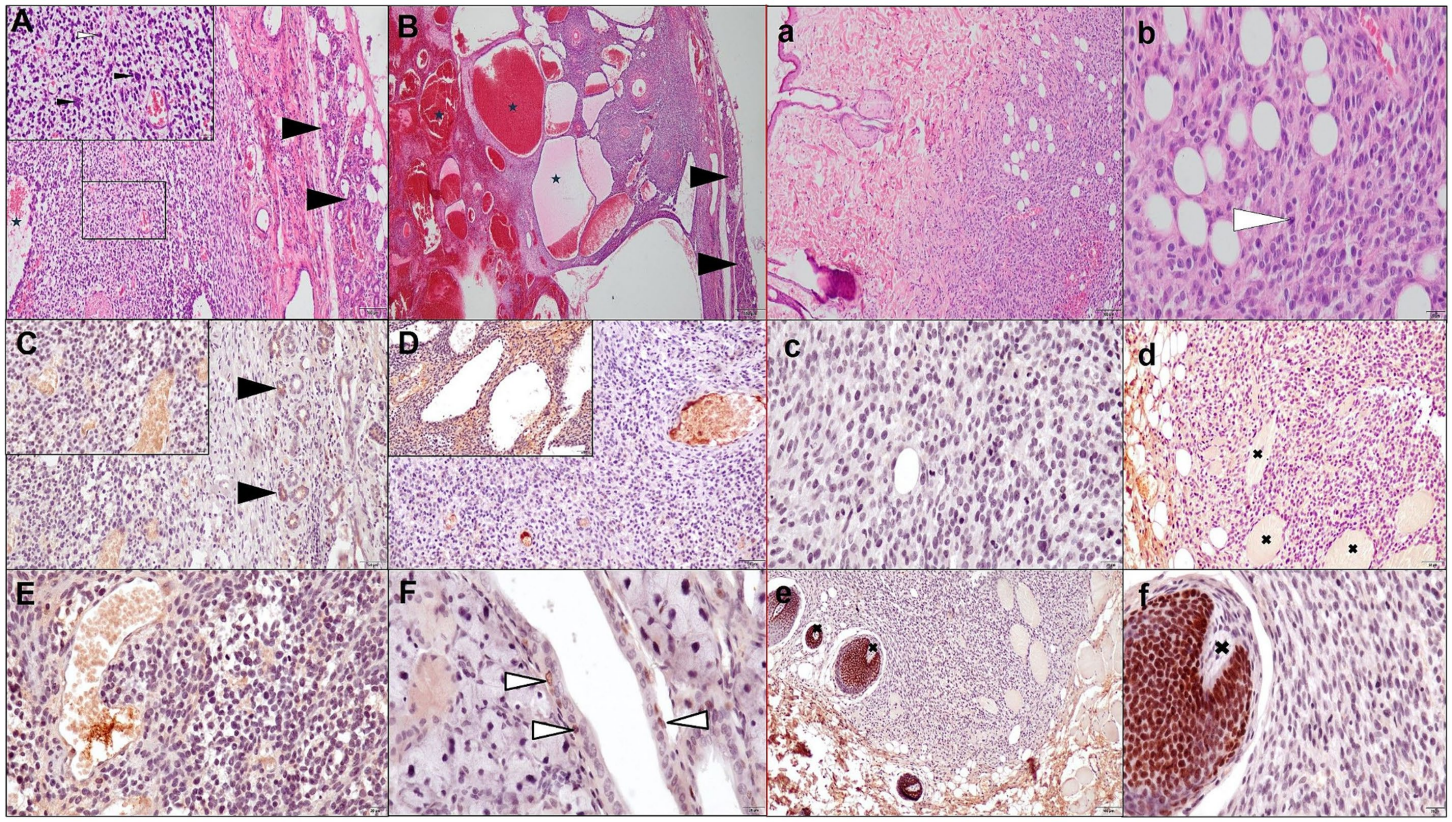
Histopathological image (hematoxylin-eosin-HE staining) and immunohistochemical (IHC) study of a poorly differentiated sarcoma located adjacent to the left mandibular salivary gland **(A,B,C–F)** and in the surrounding skin **(a,b,c–f)** being a recurrence. **(A,B)** Show a poorly demarcated, non-encapsulated tumor-rich cell neoplasm located near the salivary gland (black arrowhead) with loosely arranged neoplastic cells with indistinct cell borders, forming cystic vessel-like spaces filled to varying degrees with erythrocytes and eosinophilic fluid (asterisk). A pleomorphic tumor cells (oval, round, polygonal shape) with high anisokaryosis, a sparse cytoplasm, multinucleated cells present (inset: black thin arrowhead), and mitotic figures (white arrowhead) up to 4 in 10 HPF fields of view (2.37 mm^2^),; sparse pale connective tissue with areas of hemorrhage. Immunohistochemical examination confirmed the mesenchymal origin of the neoplastic cells: negative cytoplasmic reaction for pan-cytokeratin (**C**, arrowheads indicate CK-positive salivary epithelial cells); weakly positive cytoplasmic reaction for vimentin, although it was negative in some areas **(D)**; no nuclear reaction for p63 protein in tumor cells **(E)**. Nuclear p63 reaction in salivary myoepithelial cells is indicated by white arrowheads **(F)**. **(a,b)** Show a poorly demarcated, non-encapsulated tumor-rich cell neoplasm arranged in short interlacing streams, located in the dermis and subcutaneous tissue, infiltrating muscle fibers and hair papillae (marked with a black shout in **d–f**). Pleomorphic tumor cells (oval, spindle, round, polygonal shape) with high anisokaryosis, visible 1–2 nucleoli, moderate slightly eosinophilic cytoplasm. Numerous mitotic figures (19/10HPF, [2.37 mm^2^]) one of them indicated by a white arrowhead **(b)**. The stroma is scanty with areas of hemorrhage, but less extensive compared to the poorly differentiated sarcoma adjacent to the salivary gland. As in the sample of poorly differentiated sarcoma adjacent to the salivary gland, CK-negative **(c)**, Vim-positive to negative **(d)**, and p63-negative **(e,f)** neoplastic cells are shown. Hair matrix cells show nuclear positivity toward p63 **(e,f)**.

Three weeks after the initiation of radiotherapy no recurrence was observed in both palpation and ultrasound examinations. However, another 4 weeks later (2.5 months after surgery), a considerable deformity could already be palpated, covering almost the entire ventral region of the neck. The lesion was solid, hypoechoic, multilobate, and moderately vascularized on ultrasound examination. It measured 25.2 × 12.8 mm. Its dorsal part was poorly demarcated from the surrounding tissues. In addition, multiple cysts of varying sizes, filled with echogenic fluid, were found. The tumor was located adjacent to the external jugular vein and the left common carotid artery. Due to the suspicion of tumor recurrence and poor prognosis, euthanasia was suggested, but the owner opted for another surgery, which was performed ahortly thereafter. Histopathological examination revealed a poorly differentiated sarcoma in the salivary gland’s immediate vicinity (Figure 2).

Four days after surgery, an extensive deformity reappeared in the exact location. The overlying skin was thickened and ulcerated. Local recurrence was suspected, along with tumor infiltration of the skin. The owner decided to do a third and fourth (10 days apart) surgical removal of the tumor. During the third surgery, a hemostatic sponge with bleomycin was left in the wound, while the subsequent surgery included intratumoral administration of doxorubicin.

Histopathological examination of the tumor and skin sections again revealed poorly differentiated sarcoma (Figure 2). An immunohistochemical (IHC) study of these lesions was performed. Monoclonal antibodies against cytokeratin (CK MNF116), vimentin (Vim), p63 protein (myoepithelial cell marker), and the EnVision™, Peroxidase/DAB system (Dako, Agilent Tech. Santa Clara, CA, United States) were used. The positive control was the canine mammary gland. CK and p63 expression were absent in the tumor cells, Vim expression was negative to weakly positive in some areas. Due to financial constraints, performing further IHC differential diagnostics was impossible, e.g., for cutaneous vascular tumors.

**Figure 3:**
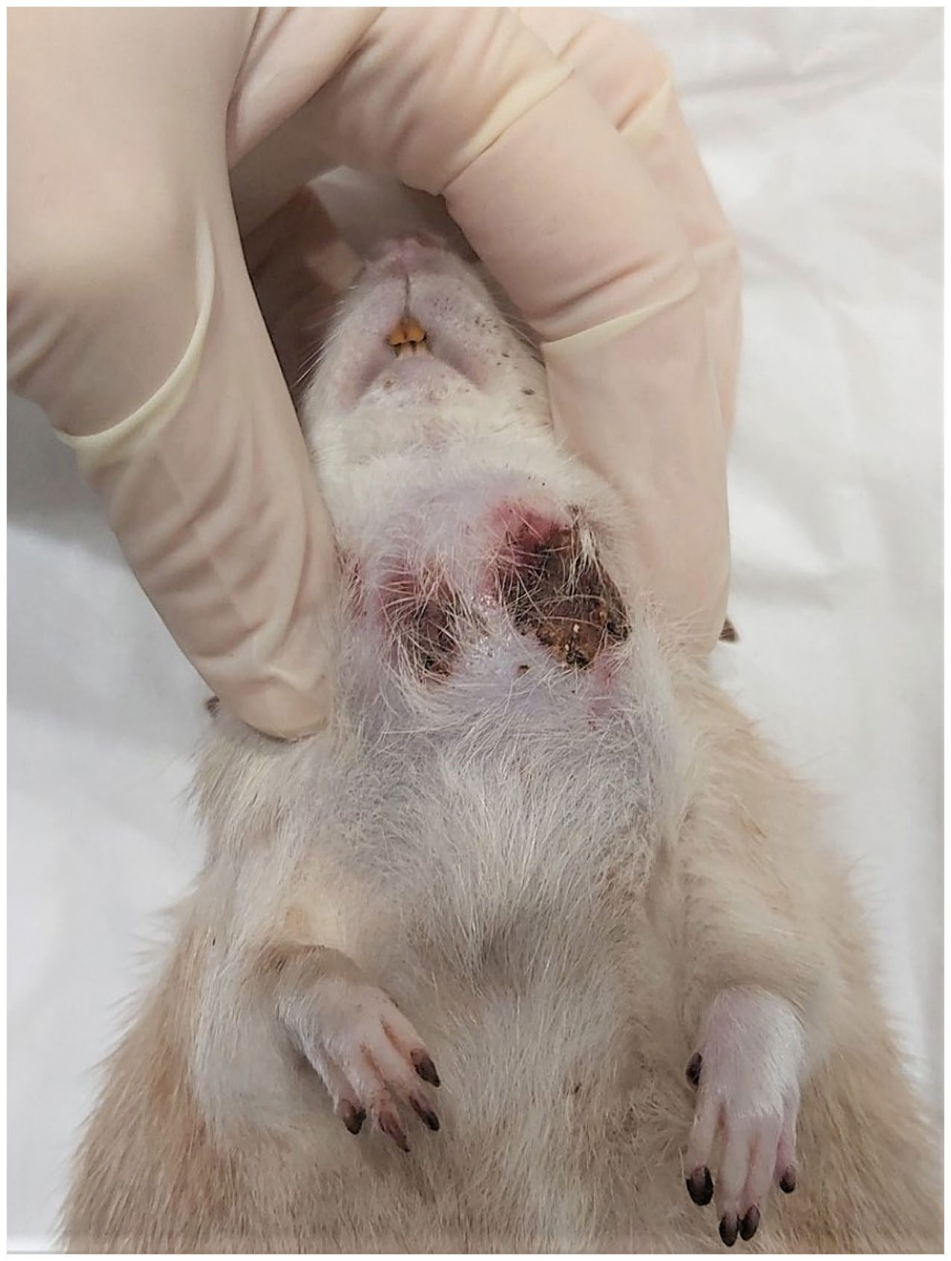
Appearance of the tumor on the day of euthanasia.

After the next 10 days, a considerable deformity measuring 25 × 12 mm was found again. The skin was covered with a wound from which bloody fluid was oozing out (Figure 3). Finally, euthanasia was performed as the rat was scratching intensely in this area, which led to bleeding. The patient also had reduced appetite and showed lower activity. The post-mortem examination revealed a nonencapsulated tumor, light pink in color, adjacent to the left external jugular vein, the left common carotid artery, and it penetrated deep into the soft tissues of the neck. No distant tumor metastases were found macroscopically.

## Discussion

The anatomy of the neck is complex mainly due to the multitude of soft tissues present in this region (1, 6). Several internal organs can be found here: salivary glands, lymph nodes, thyroid and parathyroid glands, esophagus, larynx, and trachea. In a rat, the first pair of mammary glands is also present (6). Besides the organs, the neck consists of muscles, ligaments, fasciae, vessels, nerves, and a vertebral column.

The salivary glands are represented by large and symmetrically lying clusters of glandular tissue: the paired sublingual, mandibular, and parotid glands. The mandibular salivary gland in a rat is located on the ventrolateral surface of the neck and extends from the angle of the mandible nearly to the thoracic inlet. It is oval in shape and reaches an average size of 10 × 15 × 5 mm. The four-lobed parotid gland borders it laterally and extends to the base of the external ear. The minor sublingual salivary gland is adjacent to its rostral part (6–8). Extraction of the lower incisors led to the enlargement of the mandibular and sublingual salivary glands due to hypertrophy and hyperplasia of its lobules. Rats show testosterone-dependent sexual dimorphism in the size of the mandibular salivary glands. Therefore, androgens, drugs that block their action and castration, may affect the size of this organ (7, 8). Salivary glands are relatively difficult to distinguish on palpation from the surrounding tissues.

The most common infectious agent capable of causing unilateral or bilateral inflammation of the mandibular and parotid glands, as well as the lacrimal glands and mandibular lymph nodes, is Sialodacryoadenitis virus (SDAV). The whole ventral neck region is usually swollen, which may also apply to the head. Rat coronavirus, polyomavirus, and cytomegalovirus infections usually progress without significant changes in the salivary glands (7, 8). In contrast, infection with *Klebsiella aerogenes* and *Staphylococcus aureus* can lead to abscess formation (8). As a consequence of trauma, the presence of salivary stones and foreign bodies blocking the outflow of saliva, mucocele and sublingual salivary cysts can form. However, these are rare in rats (7, 8).

The same refers to spontaneous and primary salivary gland tumors (8–11). According to Tsunenari et al., their incidence ranges from 0.02 to 0.8% (12). Cases of adenocarcinomas ranging from poorly to well differentiated (7, 9, 13), poorly differentiated carcinomas (9, 11–15), epithelial-myoepithelial carcinomas (9), myoepitheliomas (8, 9, 16, 17), or squamous cell carcinomas (7, 12) have been described in the literature. Tumors of mesenchymal origin - fibrosarcomas, undifferentiated sarcomas (7), and malignant peripheral nerve sheath tumors (7, 12) have all been found in rat salivary glands. In addition, the salivary glands can be involved in a neoplastic process in the course of leukemia (7). It is known from human medicine that salivary gland carcinomas can develop on top of existing benign tumors and progress from low-grade to high-grade malignancies (18).

In a rat, the mandibular salivary gland is adjacent to the cervical mammary gland, which is extensive in this species. The rat’s mammary gland comprises four pairs of glands and six nipples. It is important to note that mammary tumors in older female rats are the second most common neoplasm (incidence – 32.92%) (19). They are also found in males, although much less frequently. Most of these are benign, such as fibroadenomas and adenomas, although carcinomas and sarcomas may also appear (7, 20). In addition to neoplasms, retention cysts (galactocele) containing milk, cellular debris, and sometimes inflammatory infiltration cells can be expected, mainly in older females (7).

Lymph nodes in the neck region in a rat are small, with a diameter of no more than 2–3 mm, making them undetectable on palpation in healthy animals. They may occur singly or in groups. Enlargement of lymph nodes in this area may be associated with inflammation and hyperplasia in the course of infections with pathogens, such as SDAV or *Mycoplasma* spp. (7). Lymph nodes can also be the site of tumor metastases, such as the cervical lymph nodes in case of thyroid tumor, the mandibular lymph nodes in case of schwannoma, or Zymbal’s gland carcinoma (7).

Primary lymph node neoplasms such as lymphoma are rare in rats (7). They can infiltrate the skin and subcutaneous tissue. Lymphomas should be differentiated from large granular lymphocyte leukemia, the rat’s most common type of lymphoid malignancy with an incidence of 3.75–5.42% of all neoplasms (19). It progresses with splenomegaly and hepatomegaly in the later course of the disease. Enlarged organs can easily be palpated. Massive enlargement of the mesenteric, mediastinal, and mandibular lymph nodes is also possible (7). Several round, large structures can then be palpated in both - the ventral region of the neck and the middle abdomen. The patient usually presents with dyspnea and marked leucocytosis, which may be accompanied by hemolytic anemia.

The thyroid gland in the rat is located directly behind the larynx and consists of two lobes connected by an isthmus. According to the literature, spontaneous non-neoplastic and neoplastic proliferation of the thyroid gland - adenomas and carcinomas originating from follicular and thyroid C-cells, can be expected. Some carcinomas infiltrate surrounding tissues and may metastasize to regional lymph nodes and the lungs (7, 9, 19, 21). According to Nakazawa et al., thyroid neoplasms in rats are quite common, accounting for 2.5% of males and 8.33% of females (19), but can only be palpated when they reach a significant size. There are 1–2 mm parathyroid glands associated with the cranio-lateral surface of the thyroid, which can also undergo hyperplastic and neoplastic changes (7), the latter accounting for 1.25% in males (19). The authors rarely encounter thyroid and parathyroid tumors in rats in their practice

The Zymbal gland is a modified sebaceous gland found in rodents. It consists of 3–4 lobes, located rostro-ventrally to the external ear. Its duct opens adjacent to the tympanic membrane into the lumen of the external acoustic meatus. Hyperplasia of the Zymbal gland in the rat is not a response to primary degenerative or inflammatory changes involving the gland and is considered a pre-neoplastic lesion (7). Tumors from the Zymbal gland are adenomas, papillomas, but mainly carcinomas (7). Squamous cell carcinoma can infiltrate surrounding tissues and should be differentiated from squamous cell carcinoma of the skin. Zymbal gland neoplasms are palpable as bulky, slightly mobile lesions at the base of the external ear. Larger ones often ulcerate; the tumor may be visible in the lumen of the external acoustic meatus, may contain serous contents, and may be associated with swelling (7, 22).

Localization of lesions originating from the Zymbal gland is quite characteristic and is at the base of the external ear. However, the tumor may involve the lateral neck region if significant. It should also be borne in mind that malignant tumors of the Zymbal gland can give metastases to the salivary glands and the mandibular lymph nodes (7, 20).

In addition to the organs described, the neck contains cervical vertebrae, muscles, ligaments, joints, vessels, and nerves. All these structures are covered with skin and subcutaneous tissue. Thus, in the ventral region of the neck, one can also expect to find primary tumors of mesenchymal origin such as fibromas, fibrosarcomas, myxomas, myxosarcomas, histiocytic sarcomas, lipomas and liposarcomas, haemangiomas, haemangiosarcomas, perivascular wall tumors (formerly haemangiopericytomas), rhabdomyosarcomas, tumors arising from peripheral nerve sheaths and peripheral neuroblastic tumors arising from neural crest (7). Piloleiomyoma and piloleiomyosarcoma have not been described in rats so far (7, 23).

Skin tumors of epithelial origin have also been reported in rats: papilloma and squamous cell carcinoma, basal cell tumor, adenoma and carcinoma of the sebaceous glands, trichoepithelioma, keratoacanthoma, myoepithelioma, as well as those originating from the lymphatic and hematopoietic system - cutaneous lymphoma, mast cell tumor, extramedullary plasmocytoma (7). Spontaneous osteosarcomas have been described in rats, including the vertebral column, and hence, this type of tumor might also be expected in the cervical vertebrae (24). Poorly differentiated sarcomas may have foci of bone metaplasia, which may contribute to misdiagnosis (7).

A small structure called the carotid body (carotid glomus) is found bilaterally near the internal and external carotid arteries. It comprises richly vascularized epithelial tissue and has a chemoreceptor function. The rat’s carotid body weighs 60 μg (25). Tumors arising from the glomus are described in humans mainly as benign and painful deformities. There is no literature data regarding the occurrence of this type of lesion in rats.

In addition to neoplastic tumors, abscesses should also be considered in the differential diagnosis of nodular lesions in the neck region in a rat. Most commonly, these are subcutaneous abscesses that arise from skin damage, e.g., due to aggressive behavior between cagemates. Abscesses may be fluctuant and ulcerated. They can also be located in deeper tissues and originate from the organs of the neck. Infection can spread via the hematogenous and the lymphatic routes (1, 2).

In the presented case, a nodule in the ventral region of the neck was detected by a veterinarian during a standard check-up. The neck should be thoroughly palpated during the clinical examination of each patient, although relatively small and deeply located deformities may not be detected. Ultrasound may help determine the origin and extent of a nodular lesion and perform a biopsy.

Histopathological examination initially revealed a malignant myoepithelioma. Two further examinations were performed by a different histopathologist who diagnosed a poorly differentiated sarcoma. In addition, those latter two diagnoses were supported by immunohistochemistry, which ruled out the epithelial and myoepithelial origin of the tumor. However, it was not possible to confirm the vascular origin of the tumor, as indicated by multiple cavities filled with blood and proteinaceous fluid. Suspicion arose that the first diagnosis showing a malignant neoplasm was only a preliminary one, based on H-E examination alone, and it was still insufficient without immunohistochemistry.

Malignant myoepitheliomas are rare, diagnostically challenging biphasic tumors even for an experienced pathologist because of the spindle-shaped morphology of the cells in the malignant “sarcomatous” component, which can resemble sarcoma. On the other hand, it cannot be ruled out that the radiation therapy may have acted as an initiating factor in the development of the sarcoma. Radiation-induced soft tissue sarcomas (RISs) are a rare but potential late side effect of radiotherapy. According to the literature, rats developed mainly lymphomas and soft tissue sarcomas in the areas receiving radiation. Secondary sarcomas were observed at the edge of the irradiation field where the dose was higher. These areas are more prone to uncontrolled, disorganized repair tissue proliferation, leading to an increased risk of cell damage and mutagenesis and, therefore, the development of RIS (26, 27).

In this case, the epithelial-mesenchymal transition (EMT) should also be considered. ETM is essential in embryogenesis and wound healing but can also be involved in pathological processes such as cancer. It can be activated by chronic inflammation and immune disease as well as by repeated tissue trauma disrupting the healing of a wound (28). However, the authors believe it could have been sarcoma from the very beginning. After its surgical removal, the tumor could have recurred near the remaining mandibular salivary gland and the skin.

Low-grade sarcomas have been described in humans, dogs, cats, cattle, pigs, and horses. Clinically, they are rapidly growing, asymptomatic cutaneous or subcutaneous tumors, rarely giving metastases in dogs. However, they are considered to be locally invasive (29). Rapid growth is often accompanied by extensive necrosis (7).

Ideally, the diagnosis should be known before any treatment, including surgical intervention, is implemented. However, this is not easy to achieve in practice. A histological diagnosis can be problematic, especially for undifferentiated and poorly differentiated tumors, due to the tremendous histological heterogeneity of sarcomas and the recent change in nomenclature (23, 29). Cytopathological evaluation of a biopsy taken from the tumor may be helpful as a first step to exclude other possibilities, such as mast cell tumor (30). In human medicine, a fine-needle biopsy is not recommended for diagnosing sarcomas due to the insufficient material obtained with this procedure (31). A histopathological examination enriched by immunohistochemistry with a broad panel of antibodies is necessary to make a definitive diagnosis (23).

Treatment of soft tissue sarcomas is challenging in all animal species, including humans (29). There is a lack of information regarding the treatment of sarcomas in rats. Complete excision of the lesion with wide margins is the mainstay of treatment of metastasis-free lesions (29, 32). Incomplete excision of the tumor or leaving a narrow margin of healthy tissue, especially for poorly differentiated sarcomas, significantly increases the risk of local recurrence. In human and veterinary medicine, re-excision of surgical scar and radiotherapy is recommended (29, 30, 32). Postoperative radiotherapy can prolong the time to local recurrence (32). Perioperative chemotherapy with doxorubicin is effective in humans. It can also be considered an adjunctive treatment in veterinary medicine, though its efficacy in rats has not yet been known (30). Electrochemotherapy is a relatively new therapeutic technique that increases tumor cells’ uptake of chemotherapeutic agents such as bleomycin (33).

Due to the tumor’s location in the presented case, the possibility of maintaining safe tissue margins was slight from the beginning. Therefore, local recurrence had to be expected (29, 34). It took 2.5 months from the initial detection of the tumor to the emergence of the recurrence. When the recurrence was identified, the tumor was already approximately 2 cm in diameter. A further three surgeries, dictated by the owner’s desire to continue the treatment, did not have a long-term effect, as the tumor grew back quite quickly each time.

It should be noted that the patient was in excellent condition most of the time, and his owner reported no distressing symptoms, including pain. Pathological lesions in the neck can induce pressure on the trachea and esophagus, leading to dyspnea, coughing, difficulty swallowing, and regurgitation. After each surgery, the rat awoke quickly and without complications. The recovery period was uneventful, and the patient did not require a protective collar. During treatment, the patient’s other problems were adequately addressed an echocardiogram and abdominal ultrasound were performed, pneumonia was treated with antibiotics, and diabetic symptoms were controlled with insulin administration.

## Conclusion

The presented case was a starting point for considerations on the differential diagnosis when a nodular lesion is found in the rat’s ventral region of the neck. The authors’ experience indicates that it usually appears to be a tumor or an abscesses. However, because the neck anatomy is complex, there are many possibilities when it comes to determining its origin. The neck region should be thoroughly palpated during clinical examination of any patient, especially its ventral part. This case also demonstrates how complicated the diagnostic and therapeutic process can be when a nodular lesion is found in the location mentioned above. The main conclusion from this case report is to increase the awareness of veterinary pathologists and oncologists that the final diagnosis should always be part of a broad-spectrum differential diagnosis combining histopathological and immunohistochemical data, including clinical and treatment history.

Diagnosing a malignant tumor in a rat does not necessarily mean a verdict, especially when in conjunction with a dedicated owner. With advances in human and veterinary medicine, new treatment options are emerging. However, their outcomes may have not yet been reported in rats and remain in the field of experimental medicine.

## Data availability statement

The original contributions presented in the study are included in the article/supplementary material, further inquiries can be directed to the corresponding author.

## Ethics statement

Ethical approval was not required for the studies involving animals in accordance with the local legislation and institutional requirements because this study used client-owned animal. Written informed consent was obtained from the owners for the participation of their animals in this study. Written informed consent was obtained from the owners of the animals for the publication of this case report.

## Author contributions

AG: Conceptualization, Resources, Writing – original draft, Writing – review & editing. ID: Resources, Supervision, Writing – review & editing. IB: Resources, Writing – original draft, Writing – review & editing. EC: Resources, Writing – original draft, Writing – review & editing. MP: Resources, Writing – original draft. KR: Writing – review & editing. KB: Supervision, Writing – review & editing.
